# Oral glutamine supplements reduce concurrent chemoradiotherapy-induced esophagitis in patients with advanced non-small cell lung cancer

**DOI:** 10.1097/MD.0000000000014463

**Published:** 2019-02-22

**Authors:** Shih-Chieh Chang, Yi-Chun Lai, Jui-Chi Hung, Cheng-Yu Chang

**Affiliations:** a Division of Chest Medicine, Department of Internal Medicine; b Department of Critical Care Medicine, National Yang-Ming University Hospital, Yi-Lan; c Division of Infectious Disease; d Division of Chest Medicine, Department of Internal Medicine, Far Eastern Memorial Hospital, New Taipei City, Taiwan.

**Keywords:** advanced non-small cell lung cancer, concurrent chemoradiotherapy, esophagitis, glutamine

## Abstract

**Background:**

Complications related to concurrent chemoradiotherapy (CCRT) such as acute radiation-induced esophagitis (ARIE) may cause significant morbidity and unplanned treatment delays in patients with advanced non-small cell lung cancer (NSCLC). We designed a prospective randomized study to assess the impact of glutamine (GLN) supplementation in preventing CCRT-induced toxicities of advanced NSCLC patients.

**Methods:**

From September 2014 to September 2015, 60 patients diagnosed with NSCLC were included to the study. Thirty patients (50%) received prophylactic powdered GLN orally at a dose of 10 g/8 h. The prescribed radiation dose to the planning target volume was 30 Gy in 2-Gy fractions. The endpoints were radiation-induced esophagitis, mucositis, body weight loss, overall survival and progression-free survival.

**Results:**

The 60 patients with NSCLC included 42 men and 18 women with a mean age ± standard deviation of 60.3 years ± 18.2 (range, 44–78 years).

At a median follow-up of 26.4 months (range 10.4–32.2), all patients tolerated GLN well. A administration of GLN was associated with a decrease in the incidence of grade 2 or 3 ARIE (6.7% vs 53.4% for Gln+ vs Gln-; *P = *.004). GLN supplementation appeared to significantly delay ARIE onset for 5.8 days (18.2 days vs 12.4 days; *P* = .027) and reduced incidence of weight loss (20% vs 73.3%; *P = *.01).

**Discussion:**

Our study suggests a beneficial effect of oral glutamine supplementation for the prevention from radiation-induced injury and body weight loss in advanced NSCLC patients who receiving CCRT.

## 1 Introduction

Concurrent chemoradiation therapy (CCRT) is one of the major therapeutic options for non-small cell lung cancer (NSCLC), especially in locally advanced disease .^[1–3]^ Despite proven treatment efficacy, CCRT-related adverse events may result in significant morbidity and even treatment-related death .^[4,5]^ Acute radiation-induced esophagitis (ARIE) is a common complication in patients with lung or esophageal cancer receiving thoracic irradiation. CCRT remains a standard treatment option for stage IIIB NSCLC patients, but it is associated with an increase of grade 3+ esophagitis .^[6,7]^ ARIE may cause significant weight loss and unplanned treatment delays.

Glutamine, one of the most abundant amino acids in many human tissues, has been demonstrated to prevent toxicity from chemotherapy and radiotherapy .^[8]^ There have been few randomized trials regarding the effect of oral glutamine supplement on the incidence of ARIE in advanced NSCLC patients treated with CCRT. Therefore, we designed a prospective randomized study to assess the impact of glutamine supplementation in preventing CCRT-induced esophageal toxicities in advanced NSCLC patients.

## 2 Materials and methods

This study was conducted at Far Eastern Memorial Hospital, a 1200-bed tertiary referral medical center in northern Taiwan. Patients with advanced NSCLC(stage IIIB-IV by AJCC 7th edition staging), who clinically met the indication for CCRT were enrolled in the study after informed consent was obtained .^[9]^ The exclusion criteria included Eastern Cooperative Oncology Group (ECOG) performance status ≥ 2, impaired major organ functions, symptomatic brain metastasis, and life expectancy of less than 3 months .^[10]^ The patients were randomized per block to either standard treatment with prophylactic oral glutamine supplement (pure left-handed glutamine 30 grams/day in 3 divided doses, every 5 grams of powder mix with 300 ml of cold water or drink below 40°C) or standard treatment. The randomization was carried out in a 1:1 ratio and using computer-generated random numbers. All others including volunteers, study staff, investigators, and data analysts were blinded to the group assignment. There was no crossover and all subjects were analyzed in an intention-to-treat manner. Enrollment was complete following recruitment of the prespecified number of subjects. The standard treatment included 2 cycles of concurrent platinum-based doublet regimen and radiotherapy. The patients who enrolled to prophylactic oral glutamine supplement group received glutamine for 1 year. The prescribed radiation dose to the planning target volume was 30 Gy in 2-Gy fractions. Body weight change was measured after completion of CCRT. Weight loss is defined more than 5% change .^[24]^ The primary endpoint was maximal grade of ARIE and weight change. The secondary outcome was progression-free survival. The study was approved by the Institutional Review Board of Far Eastern Memorial Hospital (IRB-103029-B).

## 3 Statistical analysis

All statistical analyses were conducted using SPSS software for Windows (version22; IBM Corporation, Armonk, NY). Data are presented as frequencies for categorical variables and by mean ± standard deviation for numerical variables. Categorical variables were compared using a chi-square test or Fisher's exact test, and continuous variables were compared using an independent unpaired *t*-test. Progression-free survival was represented by a Kaplan–Meier survival curve and calculated by a log-rank test. *P*-values of <.05 were considered to be statistically significant.

## 4 Results

**Figure 1:**
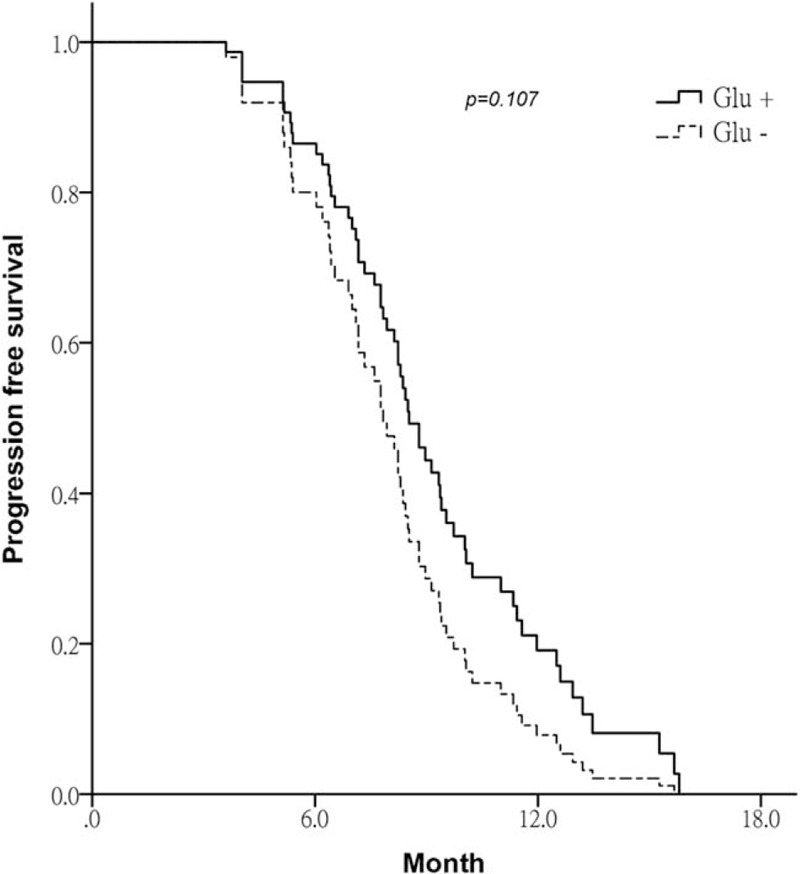
The Kaplan–Meier curve for advanced non-small cell lung cancer patients with or without glutamine administration.

From September 2014 to September 2015, 60 patients with newly diagnosed stage IIIB and IV NSCLC were enrolled in the study. Thirty patients (50%) were randomized to receive oral glutamine supplements. There were 42 men and 18 women with a mean age of 60.3 years (range, 44–78). The demographic data are summarized in Table 1. There were no significant differences between demographic characteristics between the groups, except for a male predominance in the control group. The most common histologic type of NSCLC was adenocarcinoma (45%), followed by squamous cell carcinoma (36.7%). Patients in the oral glutamine supplement and control groups received similar chemotherapy regimens of taxane/platinum, pemetrexed/platinum, and vinorelbine/platinum. After a median follow-up period of 26.4 months (range, 10.4–32.2), all patients tolerated the 12-month oral glutamine supplements well. No grade 4–5 ARIE was observed in either group. Compared with the control group, the oral glutamine supplement group had significantly less grade 2–3 ARIE (6.7% vs 53.3%; *P = *.004). In addition, oral glutamine supplements significantly delayed the onset of grade 2–3 ARIE (18.2 vs 12.4 days: *P = *.027) and reduced the incidence of weight loss (20% vs 73.3%; *P = *.01). No statistically significant differences in progression-free survival were observed between the glutamine supplement group and the control group (8.4 months vs 7.2 months, respectively; *P = *.10) by the Kaplan–Meier survival curve and log-rank test (Fig. 1). The treatment outcomes are summarized in Table 2. The median body weight change was a 1.2 kg gain in oral glutamine supplement group and a 4.6 kg loss in the control group (*P* < .001) (Table 2).

**Table 1:**
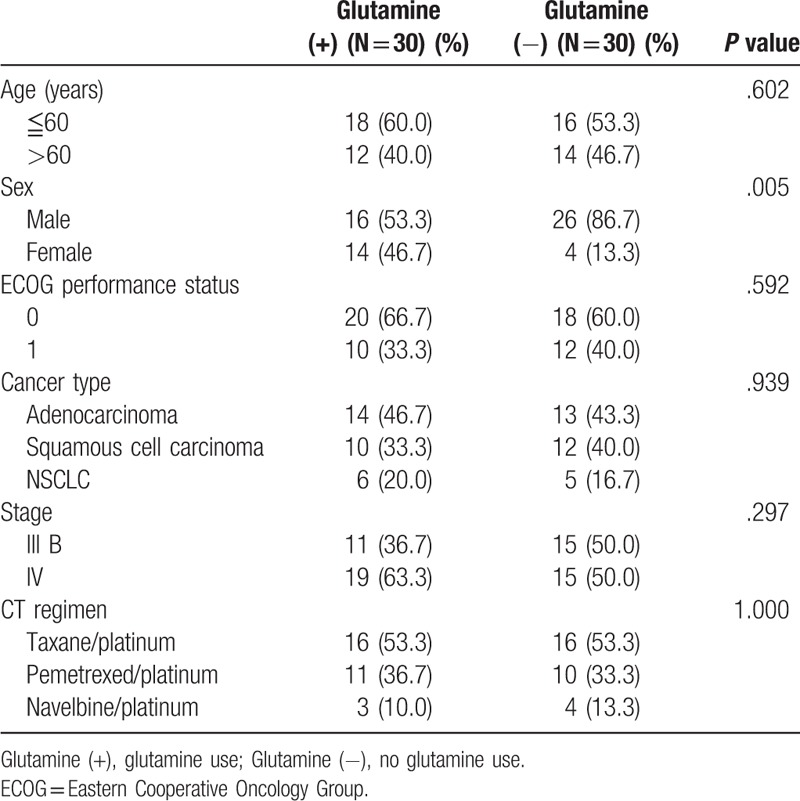
Patient characteristics.

**Table 2:**
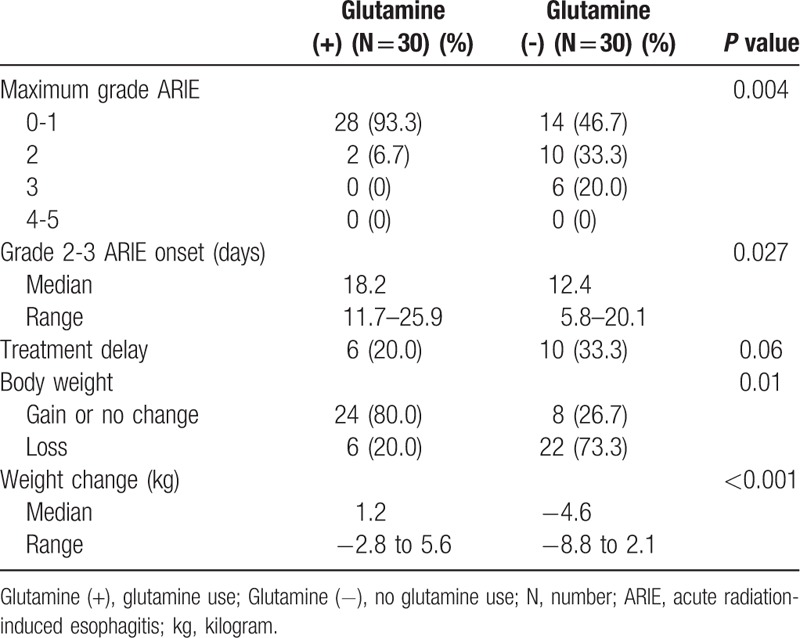
Effects of glutamine use.

## 5 Discussion

Our prospective, randomized, study demonstrated that oral glutamine supplements (L-glutamine 30 g/day in 3 divided doses) could decrease the development of severe ARIE, delay grade 2–3 ARIE onset time, and reduce the incidence of weight loss in patients with stage III–IV NSCLC who received CCRT. Glutamine is a radio-protective agent ^[8,11]^ and several studies have reported that lung cancer patients undergoing CCRT, who are taking prophylactic glutamine supplements, have a reduced risk of ARIE .^[12–17]^ The ARIE toxicity grade, weight loss, and esophageal transit times were significantly improved in the glutamine supplement group in lung cancer patients with CCRT .^[13]^ Only 2 small patient number studies showed the reducing ARIE of advance lung cancer patients undergoing radiotherapy with glutamine supplements .^[13–14]^ For unresectable stage III NSCLC, CCRT remains the standard treatment and may offer a curable opportunity with approximately 15% 5-year survival rate. Compared to sequential radiochemotherapy, CCRT have an advantage in overall survival but also increase the risk of severe acute esophageal toxicity from 4% to 18% .^[25]^ Oral glutamine supplements may reduce the incidence of adverse effect from esophageal injury and facilitate these patients to complete defined CCRT (Table 3).

**Table 3:**
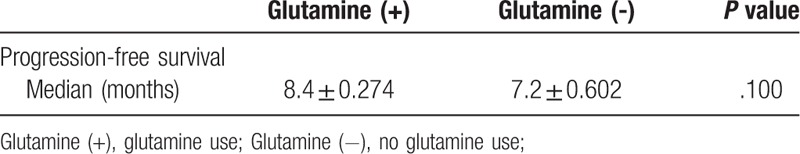
Progression-free survival based on glutamine use.

Acute radiation esophagitis often lead to dysphagia and odynophagia due to mucosal changes and effects on esophageal motility .^[18,19]^ ARIE was not only related to body weight loss, poor nutrition, and poor quality of life but also decreased the effective treatment dose of radiotherapy, increased unplanned treatment course delay, causing decreased tumor control and survival rate .^[20]^ Late radiation-induced esophagitis usually refer to onset more than 3 months after the completion of thoracic irradiation. Some patients experienced severe esophageal stricture and required surgical interventions .^[26]^ A retrospective study demonstrated prophylactic oral glutamine supplementation reduced incidence and severity of both acute and late radiation induced esophagitis .^[20]^ Body weight loss is an important prognostic factor of survival in patients with lung cancer .^[21]^ Glutamine is a neutral amino acid, which can provide host energy reserves, nutrition support in cancer cachexia, and improve radiation therapy-induced esophagitis in patients with head and neck neoplasia .^[13,22]^ Glutamine is also an oxidative supply for epithelial integrity maintenance .^[8,23]^ Several studies showed that lung cancer patients receiving glutamine supplements had significant weight gain compared with non-glutamine groups.^[13,16]^ Our study demonstrated the same result.

Our study has some limitations. First, the sample size was small and it was a single-center experience. However, the only two prospective studies for advance lung cancer patients undergoing radiotherapy and who received glutamine supplements to reduce ARIE had 32 cases ^[13]^ and 46 cases .^[14]^ Another retrospective study that enrolled advanced lung cancer patients undergoing CCRT, who received glutamine supplements had 102 cases, and another retrospective study ^[16]^ for patients with stage III lung cancer undergoing radiotherapy had 41 cases. Our prospective, randomized study included 60-advanced lung cancer cases, which is the largest study focusing on ARIE in current literature. Second, although we provided glutamine to reduce ARIE, we did not record daily calorie-intake amounts in all the patients. Third, our study focused on the relationship between oral glutamine and esophagitis, the palliative CCRT of our patients including stage IIIB and stage IV were by clinical decisions. Forth, the radiation regimen is not the standard prescription doses for CCRT in advanced NSCLC. We use the 30 Gy in 2-Gy fractions as a palliative CCRT to minimize the radiation injury for Asia patients. According to the American Society for Radiation Oncology (ASTRO) guideline,^[27]^ a moderately hypofractionated approach with external beam radiation therapy is recommended, where “moderate” is defined as daily radiation doses of 280–300 cGy) per fraction to a total dose of 3000 or 4200 cGy. Higher radiation doses may not be tolerable for palliative-intent patients, and lower doses may not confer a quality-of-life benefit. The best practice also includes minimizing unnecessary radiation dose to the esophagus. Fifth, the radiation location, involved esophagus volume and chemotherapy regimen can also affect the incidence and degree of esophagitis. In our study, we design CCRT on primary tumor site and cannot distinguish the influence of radiation location and chemotherapy regimen due to small patient numbers.

## 6 Conclusions

In conclusion, this study demonstrated that oral glutamine supplements has a benefit in delaying onset of and decrease the severity of ARIE in advanced lung cancer patients undergoing CCRT. With oral glutamine use, the patients had improved quality of life and better treatment outcomes.

## Author contributions

**Data curation:** Jui-Chi Hung.

**Formal analysis:** Yi-Chun Lai, Cheng-Yu Chang, Shih-Chieh Chang.

**Methodology:** Yi-Chun Lai, Cheng-Yu Chang, Shih-Chieh Chang.

**Validation:** Yi-Chun Lai, Jui-Chi Hung.

**Writing – review & editing:** Yi-Chun Lai, Cheng-Yu Chang, Shih-Chieh Chang.
